# Enabling High-Throughput Tissue Sectioning for Robust Lipid Mass Spectrometry Imaging

**DOI:** 10.1021/acs.analchem.6c03106

**Published:** 2026-09-22

**Authors:** Ruben Jacobs, Ritesh Chidambaram, Vincent Steinbacher, Chenwei Wei, Tim Ragan, Michiel Vandenbosch, Ron M. A. Heeren

**Affiliations:** † The Maastricht MultiModal Molecular Imaging (M4I) Institute, Division of Imaging Mass Spectrometry (IMS), 5211 Maastricht University, Maastricht 6229 ER, The Netherlands; ‡ TissueVision 150 California St STE 303, Newton, MA 02458, The United States; § Institute of Molecular Immunology, School of Medicine and Health, Technical University of Munich (TUM), Munich 81675, Germany

## Abstract

Matrix-assisted laser desorption/ionization mass spectrometry imaging (MALDI-MSI) has been established as a powerful tool for the spatial characterization of molecules. Recent advances have been driven by improvements in the spatial resolution, acquisition time, and sensitivity. Although MALDI-MSI excels in the label-free, multiplexed detection of biomolecules, variations in sample preparation remain a significant source of experimental variability. Here, we present a workflow demonstrating the integration of a robust, completely automated tissue section processing system into a MALDI workflow. Importantly, PFA fixation and agarose embedding are demonstrated to preserve lipid compositional distributions. Combined, it enables robust and stable lipid imaging across a completely sectioned mouse brain. The integration of the Marinus MP system advances mass-spectrometry imaging toward a fully automated workflow, significantly reduces sample-preparation variability, and enables more consistent, reliable data acquisition and improved biological interpretation. Parallel with sectioning, serial two-photon (STP) tomography is used to acquire high-resolution digital images of every single section, providing a structural reference for downstream analyses. We demonstrate how the Allen Mouse Brain Atlas can be seamlessly integrated with these multimodal imaging data sets. This workflow enables precise region-specific comparisons and enriches the anatomical context with robust molecular information.

## Graphical abstract

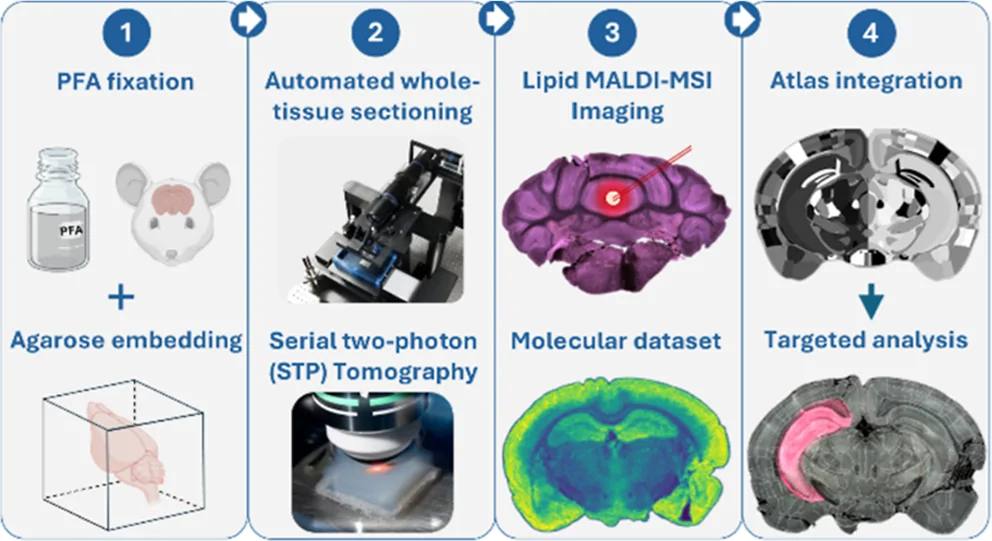

## Introduction

Matrix-assisted laser desorption/ionization mass spectrometry imaging (MALDI-MSI) is a powerful analytical tool for label-free spatial mapping of molecules in situ. The technique’s ability to simultaneously detect thousands of ions in a single experiment makes it one of the most popular and widely used MS imaging methods around.^1,2^ The potential of MALDI-MSI in assisting molecular diagnoses has been underlined by the consistent increase of MALDI-MSI papers published.^3^ Although continuous advancements have been made in spatial and spectral resolution, sensitivity, and acquisition time, the widespread translational adoption of this technique in clinical practice remains challenging. One of the main reasons that hinders MALDI-MSI from reaching its full clinical potential is related to method reproducibility, especially for processing formalin-fixed paraffin-embedded (FFPE) tissues.^4^ Thanks to its specimen-preserving properties, FFPE is the standard format for clinical samples. However, FFPE samples typically undergo extensive mechanical and chemical treatments during processing, which results in a loss of crucial endogenous molecules.^5,6^ Consequently, alternative preparation strategies that better preserve the targeted molecular content might be required for MALDI workflows. Here, an alternative embedding method is proposed in which the mouse brain tissue was fixed using 4% paraformaldehyde (PFA) and embedded in agarose.

In a routine MALDI workflow, one of the initial downstream steps after tissue embedding usually involves cutting the tissue sections. This stage is highly delicate and requires precision, as even slight inconsistencies during sectioning can have significant downstream effects.^7,8^ Such irregularities not only complicate data interpretation but also can reduce reproducibility across different regions of the same sample or between multiple samples. As a result, meticulous optimization of the tissue sectioning process is critical for achieving reliable and reproducible MALDI-MSI data sets. One effective approach that improves data consistency involves the reduction of the operator-dependent variability. Implementing automated sectioning systems that preserve tissue morphology, maintain lipid integrity, and ensure controlled storage conditions can substantially enhance the reproducibility and data quality in large-scale whole-tissue molecular studies.^9^

In this study, we aimed to improve the throughput and reproducibility of MALDI-based imaging across an entire mouse brain by employing an automated imaging and sectioning platform developed by TissueVision, Inc. (Newton, MA, USA). This system integrates high-throughput sectioning with optical imaging by allowing the combination of high-speed two-photon microscopy with state-of-the-art automated tissue sectioning.^10–12^ Through this integrated design, an entire mouse brain could be microscopically imaged and sectioned sequentially within 10 h, maintaining precise spatial correspondence between the optical images and physical sections. Furthermore, spatial lipidomics datasets acquired using MALDI-MSI were seamlessly co-registered with the corresponding two-photon microscopy data, enabling direct correlation between molecular and structural features. This data was augmented through the incorporation of spatial references from the Allen Mouse Brain Atlas,^13–15^ which was automatically aligned with the morphological and molecular data. The resulting integrated multimodal dataset allows anatomical regions to be directly compared and interpreted through their corresponding molecular signatures. Importantly, the PFA fixation and agarose embedding procedure is demonstrated to preserve the lipid compositional distributions throughout the brain.

## Methods

### Chemicals and Reagents

Ketamine (100 mg/kg), xylazine (10 mg/kg), and phosphate-buffered saline (PBS) for anesthesia were ordered from Medistar, CP-pharma, and Capricorn Scientific, respectively. 4% paraformaldehyde (PFA) was purchased from Carl Roth and diluted in PBS (Capricorn Scientific). Norharmane and *tert*-butyl methyl ether (MTBE) were obtained from Sigma-Aldrich (Zwijndrecht, NL). Indium tin oxide (ITO) glass slides were purchased from Delta Technologies (Loveland, USA). Water, methanol, and chloroform, all ULC/MS grade, were purchased from Biosolve (Valkenswaard, NL).

### Animal Experiments

Six-week-old C57BL/6J male mice were purchased from Janvier Labs (Charles River Europe). Mice were housed in pathogen-free conditions at the Animal Core Facility of the School of Medicine, TUM. Mice were housed with a 12 h light/12 h dark cycle. The temperature was set to 22 ± 2 °C, and the humidity was set to 55 ± 10%, and it was checked daily. Guidelines of the Federation of European Laboratory Animal Science Associations were implemented for experiments. Experiments were approved by the District Government of Upper Bavaria (permission nos. ROB-55.2-2532.Vet_02-18-16). Mice were anesthetized via intraperitoneal injection at a dose of 0.01 mL/mg body weight. After 10 min, animals were assessed using the toe-pinch reflex, and a supplementary dose equivalent to 25% of the initial volume was administered if responsiveness persisted. Transcardial perfusion was performed using 50 mL of phosphate-buffered saline (PBS), followed by 50 mL of 4% paraformaldehyde (PFA), delivered at a constant rate of 10 mL/min. Brains were subsequently excised and post-fixed in 4% PFA for 24 h at 4 °C. The fixative was then replaced with PBS containing 0.1% sodium azide, and samples were incubated for an additional 24 h at 4 °C. Finally, the solution was exchanged once more for fresh PBS with 0.1% sodium azide, and brains were stored at 4 °C until downstream processing. All PFA fixed/embedded data results from one single, fully sectioned mouse brain. For comparison, a second control mouse brain devoid of fixation was excised and snap frozen for downstream analysis.

### Brain Tissue Preparation

The automated imaging and sectioning procedure were performed as described before.^11^ Briefly, a standard single mouse brain embedding procedure was performed using the TissueVision 400 IMbed protocol as follows: the brain was placed in the center of an M014 mold with a pair of detection markers installed in the mold, 4.5% molten agarose was poured into the mold at 60 °C, and it was allowed to solidify. The resulting block was removed from the mold and soaked in 400 IMbed solution for 24–72 h at 4 °C. The solution was then polymerized at 40 °C for 6 h, and the resulting polymer block was extracted and placed in phosphate buffer for storage at 4 °C.

### Tissue Sectioning

The Marinus MP system was set up in the following manner: the tissue block was attached to a water bath platform and mounted in the water bath filled with room temperature phosphate buffer, which was then loaded into the system. A ceramic blade was installed for sectioning the sample, and the system was set up to collect 300 sections at a sectioning speed of 0.5 mm/s and a section thickness of 50 μm. Sections were mounted onto standard glass slides with the exception of every 10th section being captured on indium–tin oxide-coated glass slides (ITO, CG-40IN-S115, Delta Technologies, USA). The system imaging parameters were to image at 40 μm below the sectioning surface, with a resolution of 1.38 μm/pixel, at 820 nm laser excitation wavelength, collecting three color channels (red, green, and blue). Sections were then sequentially unloaded into labeled slide boxes and stored at 4 °C prior to shipping in a temperature-controlled package. Collected image data was uploaded and processed on the web-based TVI Portal. The control fresh-frozen mouse brain was sectioned at the same thickness as above using a cryotome (Leica, Rijswijk, The Netherlands) at −20 °C, followed by thaw-mounting onto ITO slides. Slides were stored at −80 °C until further analysis.

### Matrix Application

Brain tissue slides were sprayed with norharmane (7 mg/mL in MeOH/CHCl3 1:2) using an automated HTX M3 sprayer (HTX Technologies, LLC, Chapel Hill, NC). Spraying parameters were as follows: 15 layers of matrix were applied at a flow rate of 120 μL/min, with a dry time of 30 s in between passes using a track spacing of 3 mm. The spraying nozzle was heated to 30 °C, and its speed was set to 1200 mm/min, maintaining a stable spray at 10 psi.

### Mass Spectrometry Imaging

Lipid spectra were acquired in both positive and negative polarity using a RapifleX MALDI Tissuetyper in reflectron mode (Bruker Daltonik GmbH, Germany), covering a mass range of *m*/*z* 340–2000 with a spatial resolution of 50 μm × 50 μm. 200 laser shots per pixel at a frequency of 10 000 Hz. The method was calibrated using red phosphorus before each imaging run.

### Data Analysis

Data processing was performed using SCiLS lab software 2025a (SCiLS, Bremen, Germany). All imaging data was recalibrated in flexAnalysis and normalized to the root mean square (RMS). Imaging datasets were converted to open file format (imzml) and uploaded into the TVI Portal. Co-registration was performed using the TVI Portal’s automated pipeline, enabling overlays between STP and MALDI datasets. Briefly, the pipeline performs an automated registration of MALDI to STP images using a series of preprocessing and Elastix non-rigid registration operations. Both the MALDI and STP images pass through a series of preprocessing steps to normalize the appearance and isolate the tissue foreground to ensure accurate Elastix registration. The preprocessing step also extracts a number of fiduciary points from the images and is saved as a JSON payload, which will later be used for estimating the registration accuracy. Post Elastix registration, the fiduciary contour points drive the registration accuracy assessment. Fixed and moving contour coordinates are scaled to their respective pixel sizes. A nearest neighbor matching is performed to pair each fixed contour point with a moving contour point. The matched pairs then yield a mean residual distance, which serves as an accuracy measure. For qualitative validation, the matched pairs are also binned into 20 bins about the fixed image’s center of mass to produce a per-sector distance profile.

### Lipid Identification

Six unimaged brain tissue sections collected throughout the brain were scraped from microscope slides and processed for lipid extraction. For comparison, six sections from the fresh-frozen brain tissue were subjected to the identical protocol, adapted from Matyash et al.^16^ Samples were extracted by adding 375 μL of methanol and 1250 μL of MTBE, followed by vortexing for 1 min and incubation at room temperature for 60 min. Subsequently, 350 μL of HPLC-grade water was added, and samples were centrifuged at 1000 g for 10 min. The upper organic phase was transferred to a 2 mL Eppendorf tube. The remaining lower phase was re-extracted with 600 μL of MTBE, 180 μL of methanol, and 150 μL of water, vortexed for 1 min, and centrifuged again at 1000 g for 10 min. The resulting MTBE phase was collected and combined with the initial extract. Pooled extracts were dried and reconstituted in 50 μL of acetonitrile-isopropanol (1:1, v/v) prior to analysis.

LC–MS lipidomics analysis was adapted from Breitkopf et al.^17^ In brief, lipid separation was performed on a Thermo Scientific (Dionex) Ultimate 3000 Rapid Separation UHPLC system equipped with a Thermo Scientific Hypersil Gold C18 analytical column (10 cm, ID 2.1 mm, 1.9 μm). Separation was established by a 18 min gradient starting from 68% mobile phase A (ACN/H2O 60:40 (v/v), 10 mM AF) to 97% mobile phase B (IPA/ACN 90:10 (v/v), 10 mM AF) at a flow rate of 0.25 mL/min. The HPLC system was coupled to a Q Exactive HF mass spectrometer (Thermo Scientific). The MS was operated in the Data Dependent Acquisition (DDA) mode. Full MS scans were acquired between 200–1450 *m*/*z* at a resolution of 60.000, followed by MS/MS scans of the top 8 most intense ions at a resolution of 30.000, with dynamic exclusion set at 60 s. Subsequent data analysis was performed in Lipostar (v2.1.9).

## Results and Discussion

### Automated Marinus MP Sectioning Produces Uniform Mouse Brain Sections and Enables Streamlined Data Visualization

Achieving reproducible data remains a major challenge in MALDI-MSI workflows, as sample preparation critically influences tissue integrity, ionization efficiency, and analyte abundances.^18,19^ To improve standardization and minimize technical variability, the Marinus MP was integrated into the MALDI-MSI workflow (Figure 1A). The brains were fixed using a perfusion fixation with 4% PFA. Following excision, intact mouse brains were embedded in agarose prior to automated sectioning. This approach enabled uniform, time-efficient sectioning while producing consistently cut and mounted tissue sections at precisely controlled thicknesses on slide types compatible with downstream analyses. The automated sectioning procedure resulted in the complete collection of over 250 sections in under 10 h. No tissue folding or incomplete transfer to the slide occurred, and, as a result, all sections could be included for further analysis, highlighting the robustness of the Marinus MP system. In parallel with sectioning, TissueVision’s integrated serial two-photon (STP) tomography acquired high-resolution blockface images in four color channels prior to each section. The incorporation of the STP modality provided high-resolution anatomical context, reducing the need for additional hematoxylin and eosin (H&E) staining. The STP images were co-registered with the corresponding MALDI-MSI dataset (Figure 1B), and the Allen Mouse Brain Atlas was subsequently aligned to facilitate region-specific analyses (Figure 1C). The resulting imaging data were uploaded to the TVI viewer portal, allowing visualization of all individual brain sections (Figure 2).

**Figure 1:**
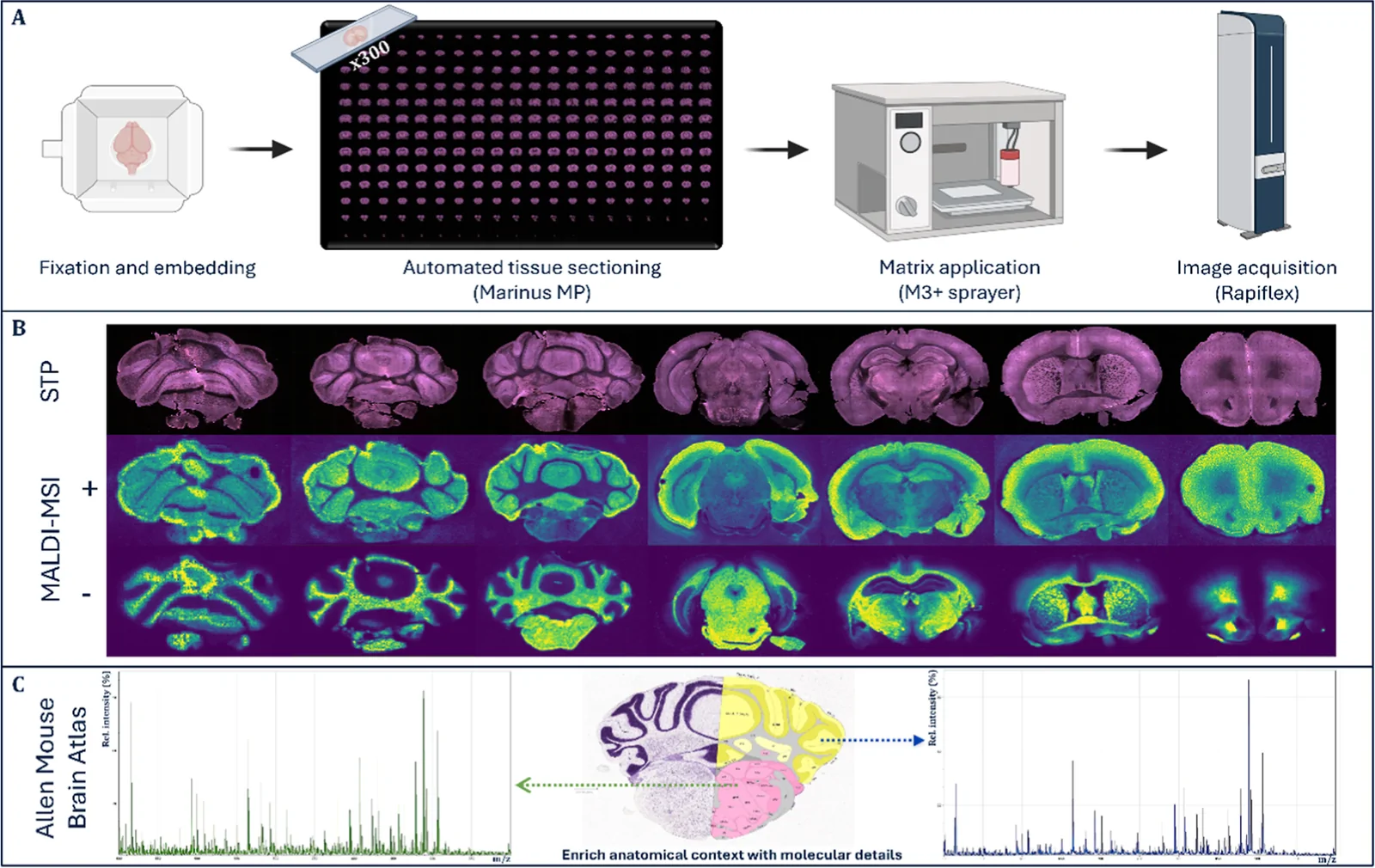
Overview of the study workflow and data integration. (A) Schematic overview of the study design. Excised mouse brains were fixed in 4% paraformaldehyde (PFA), embedded in 4.5% agarose, and serially sectioned and imaged using the automated Marinus MP system. Sections were processed following a standard MALDI–MSI lipid workflow, using norharmane as a matrix. Acquisition in both polarities on a Rapiflex at 50 μm spatial resolution. Images made in Biorender. (B) Overview of data generation and co-registration. Block-face serial two-photon (STP) images acquired by the Marinus MP system were loaded into the TVI viewer. MALDI–MSI data were subsequently co-registered to the corresponding STP dataset. Representative images shown. (C). Atlas-based anatomical integration. The Allen Mouse Brain Atlas was morphed onto each individual section, enabling region-specific molecular analysis and targeted comparisons across brain structures. Brain image imported directly from the Allen Mouse Brain Atlas.

**Figure 2:**
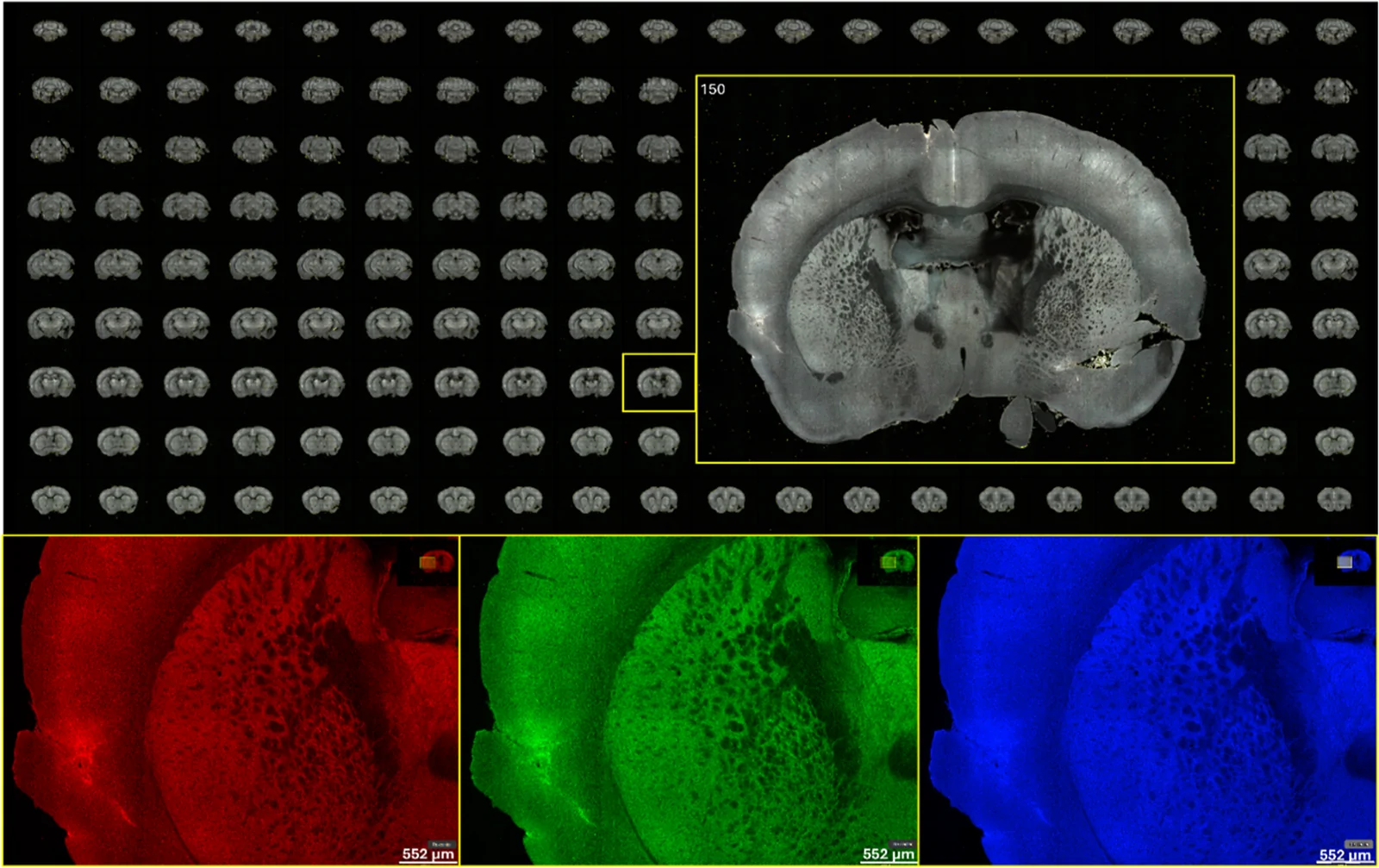
TVI viewer visualization of Marinus MP’s block-face imaging data. Block-face serial two-photon (STP) images acquired using the Marinus MPs system were loaded into the TVI viewer software for visualization and downstream integration. All sections can be viewed simultaneously, or for higher resolution a section of interest can be selected (here, Section 150). Images were acquired in three autofluorescence channels with a resolution of 1.38 μm/pixel at 820 nm laser excitation wavelength.

### Consistent Lipid Imaging Throughout a Fully Sectioned Murine Brain Using MALDI-MSI

The non-destructive nature of the STP tomography procedure enables the generation of high-quality tissue sections, making them well-suited for investigating underlying biomolecular signatures.^10,11^ Given the reproducibility challenges commonly encountered in MALDI-MSI, we evaluated whether the sample preparation procedures needed for the Marinus approach could be robustly integrated into the MALDI-MSI workflow while maintaining lipid detectability. For this purpose, every 10th section was captured on conductive ITO slides. The resulting 21 ITO-mounted tissue sections were sprayed with norharmane and subsequently imaged on a RapifleX MALDI-TOF Tissuetyper (Bruker Daltonics, Bremen, Germany). The average spectrum obtained and the corresponding spatial distribution of selected identified lipid species for each polarity are shown in Figure 3.

**Figure 3:**
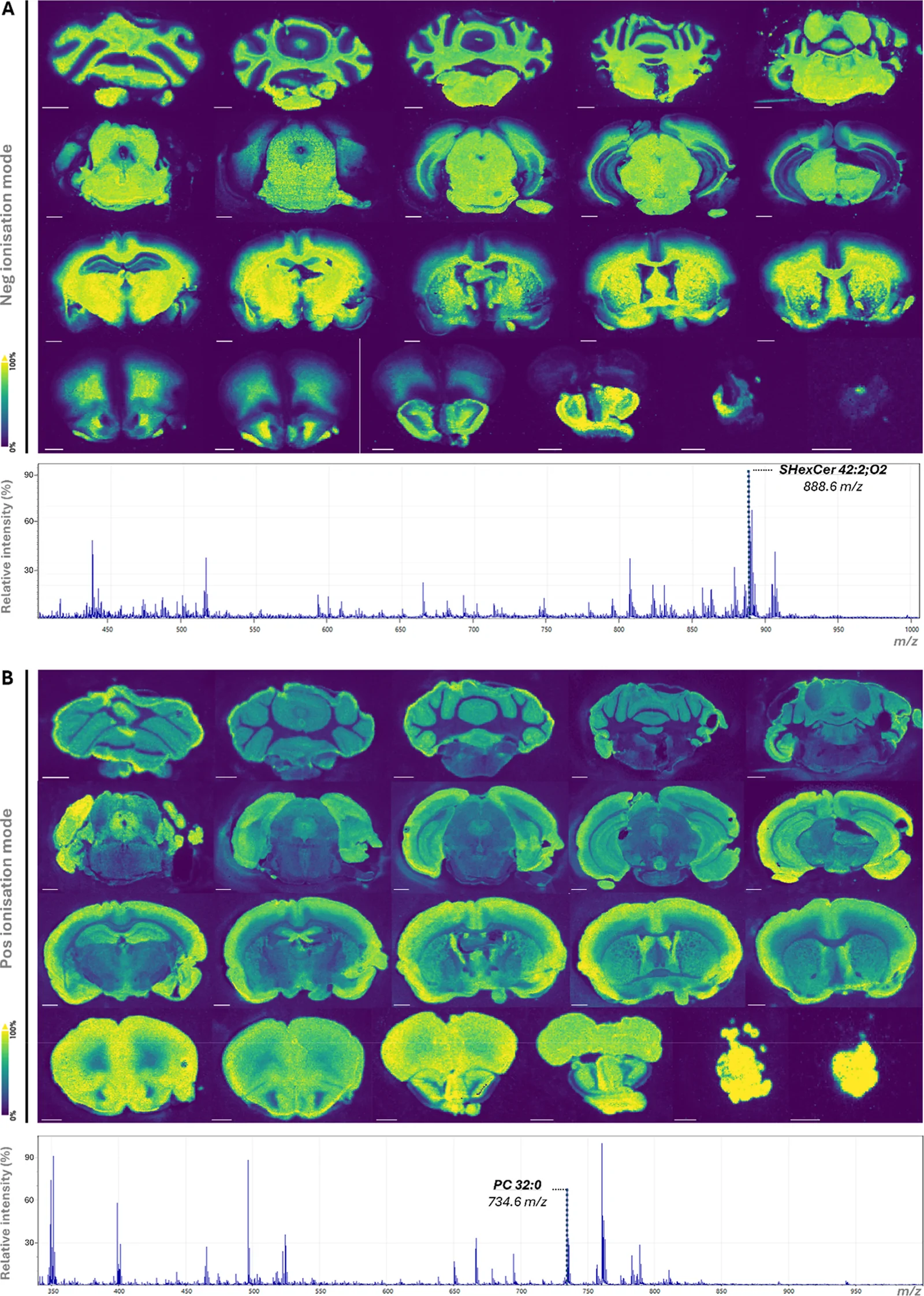
Marinus MP platform enables robust MALDI-based imaging across the entire brain. (A) Spatial distribution of *m*/*z* 888.6, identified as SHexCer 42:2; O_2_ shown across the mouse brain. The corresponding average mass spectrum (RMS normalized) acquired on a Rapiflex in negative ion mode is shown. (B) Spatial distribution of *m*/*z* 734.6, identified as PC 32:0 shown across the mouse brain. The corresponding average mass spectrum (RMS normalized) acquired on a Rapiflex in positive ion mode is shown. All scale bars are set at 1 mm.

Six brain sections that were not used for mass spectrometry analysis were scraped off the slides and used for lipid identification using LC–MS to further identify the measured *m*/*z* values. Across the brain, we successfully identified 355 lipid species across both polarities (Supporting Information Data S1). To assess the impact of the used sample preparation workflow on the lipid profile, an additional fresh frozen brain was manually sectioned, sprayed, and measured. A comparison between the fresh frozen and fixed/embedded brain sections from the Marinus platform is shown in Figure 4A,B. Considering lipid intensities, a moderate overall reduction was observed across most lipid classes in the positive ionization mode (normalized intensity ratio of the PFA fixed tissue compared to fresh frozen tissue <1). In contrast, in negative ionization mode, several lipids, including sphingolipids (e.g., PC 42:7, SHexCer 42:1/42:2/36:1, etc.) and glycerophospholipids (PC 40:6, PC 42:7, etc.), showed comparable or higher abundances (ratio>1) relative to the fresh frozen measurement. Overall, the proposed sample preparation protocol does not appear to compromise overall lipid detectability, as a broad range of lipid species remain detectable. Lipid intensities seem to be slightly affected predominantly in the positive ionization mode. To validate the MALDI-MSI results, six fresh frozen sections were captured and subjected to LC–MS analysis. The LC–MS results confirm the imaging results by indicating a moderate loss in lipid intensities and identifications (−17%) in the positive ion mode. The amount and intensities of negatively charged lipids remain comparable between fresh frozen and PFA-fixed/agarose-embedded tissues. Remarkably, the LC–MS analysis identified eight additional lipids in the fixed brain (Figure 4C). To assess the impact of the fixation and embedding on analyte delocalization, an additional high spatial resolution (7 μm) measurement on the timsTOF (Bruker Daltonics) was performed, where a fixed section was compared to its fresh frozen counterpart (Supporting Information Figure S1). Analyte localization was not visibly altered due to the fixation workflow. Furthermore, these results support the conclusions drawn from the 50 μm measurements, indicating that broad lipid coverage is maintained. These findings indicate that the PFA fixation and agarose embedding procedure have a minimal effect on the lipid profiles, rendering the proposed sample preparation suitable to study intact lipid distributions by MALDI-MSI. Furthermore, the elimination of the extensive washing steps required for FFPE tissues allows the proposed workflow to retain native lipid profiles, in contrast to FFPE-based approaches.

**Figure 4:**
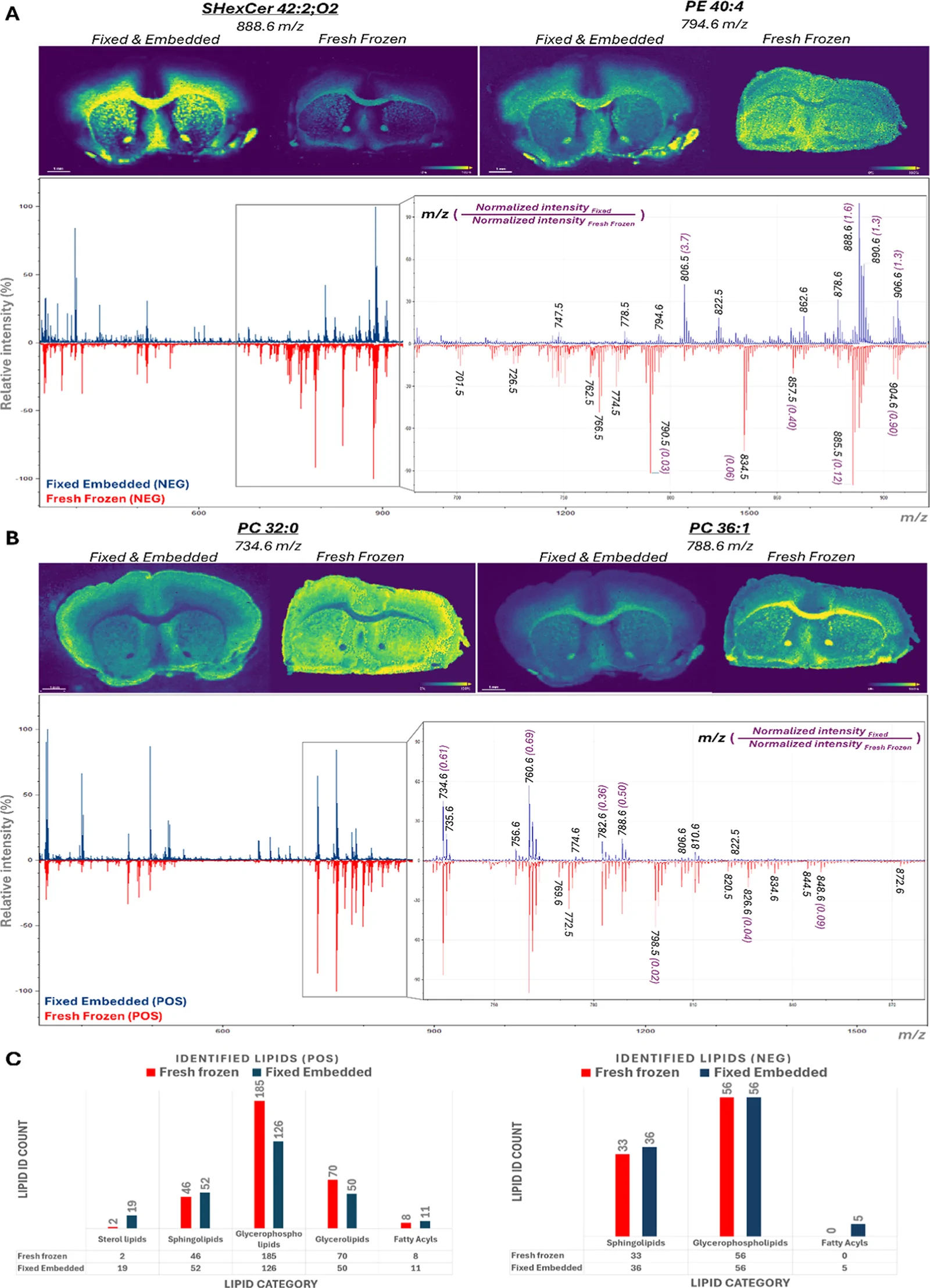
Impact of fixation and embedding on lipid profiles. (A) Spatial distribution of selected lipids in coronal mouse brain sections using MALDI-MSI in the negative ion mode on the Rapiflex. Ion images of 888.6 *m*/*z* and 794.6 *m*/*z* corresponding to SHexCer 42:2; O2 and PE 40:4, respectively, are shown. Related spectra from fresh frozen and fixed/embedded tissue were RMS normalized. The lipid mass range was enhanced, and the most abundant species were annotated by *m*/*z*. (B) Spatial distribution of selected lipids in coronal mouse brain sections using the MALDI-MSI in positive ion mode on the Rapiflex. Ion images of 734.6 *m*/*z* and 788.6 *m*/*z* corresponding to PC 32:0 and PC 36:1, respectively, are shown. Related spectra from fresh frozen and fixed/embedded tissue were RMS normalized. Lipid mass range was enhanced, and the most abundant species were annotated by *m*/*z*. (C). Comparison of the identified lipid compounds in fresh frozen versus paraformaldehyde fixed and agarose-embedded tissues. A complete overview of identified lipid species is shown in Supporting Information Data S1.

To further explore the capabilities of the slide set generated by the Marinus sectioning workflow, we assessed its suitability for MALDI-MSI at higher spectral and spatial resolutions. Additionally, the use of an elevated-pressure MALDI system allowed the evaluation of imaging brain sections captured on nonconductive slides. Accordingly, MALDI-MSI experiments were performed on both an FT-ICR (Solarix) system to achieve ultrahigh spectral resolution and a timsTOF fleX instrument for higher spatial resolution, adding to the dataset acquired on the RapifleX. Complementary mass-spectrometry datasets are provided in Supporting Information Figure S2, illustrating the robustness of the sections across different MSI platforms.

### Integrating MALDI-MSI with the Allen Mouse Brain Atlas for Region-Specific Molecular Profiling

Understanding how molecular species are distributed across anatomical regions is fundamental to deciphering biological processes within the tissues. MALDI-MSI excels in the label-free spatial characterization of a wide range of biomolecules. Its specialized sample preparation and comparatively limited spatial resolution relative to microscopy underscore the need for complementary imaging modalities.^20^ Traditionally, MSI data are interpreted through alignment with conventional histological stains such as H&E, which provide valuable morphological context but capture only a single structural snapshot of the tissue.^21^ The TVI Portal was developed to integrate multiple imaging modalities within a unified visualization environment to overcome these limitations and embed molecular information into a robust anatomical framework. In this workflow, en face STP tomography images serve as an undistorted reference frame to which secondary analyses can be registered back into the correct CCFV3 ontology space.^10,15^ The image alignment strategy is automated, allowing precise overlap of multimodal data sets. The accuracy of alignment can be visualized for each individual section by the per-sector distance profile (Supporting Information Figure S3). In addition, the TVI Portal allows seamless overlay of the Allen Mouse Brain Atlas, facilitating precise region-specific comparisons and direct association of molecular profiles, such as lipid distributions, with defined brain structures (Figure 5). While the alignment of the Allen Brain Atlas to MSI data has been reported before, our workflow eliminates the need for prerequisites such as histological stains for the registration to work.^22^

**Figure 5:**
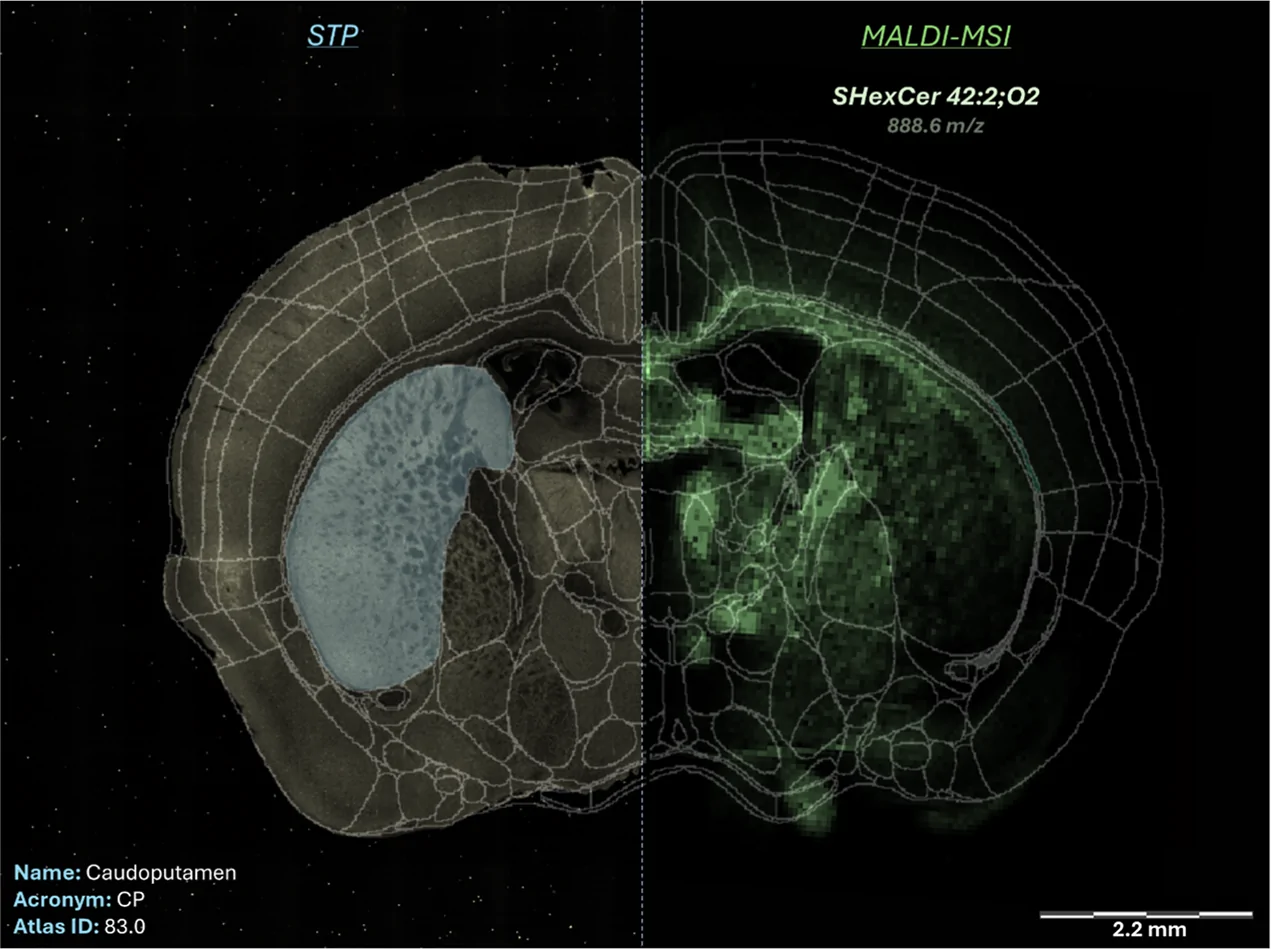
Multimodal data integration. Integration of serial two-photon (STP) tomography and MALDI datasets with the Allen Mouse Brain Atlas is shown. The atlas is morphed onto the tissue to enable regional annotation, with the caudoputamen highlighted. Individual datasets can be toggled on and off to facilitate region-specific comparisons.

The inclusion of 3D visualization and registration capabilities extends this analysis by enabling whole-brain mapping of specific lipid signatures, revealing spatial patterns across anatomical regions that are difficult to capture in two-dimensional analyses or on individual manually cut sections that lack an en-face registration target. This study demonstrates the integration of STP and MALDI-MSI data but is not limited to these two modalities. The implemented platform readily supports additional modalities, including immunofluorescence microscopy, spatial transcriptomics, and clinically relevant techniques such as magnetic resonance imaging (MRI). Ultimately, the integration of complementary spatial datasets across molecular and anatomical scales provides a more holistic understanding of tissue organization and function.

## Conclusion

The ability to acquire both morphological and molecular information from the same sample in a time-efficient and standardized manner represents a significant step forward in large-scale tissue-imaging studies. By minimizing manual handling and variability in sectioning quality, the automated approach enhances reproducibility, preserves tissue integrity, and ensures a consistent section thickness across the entire organ. This provides a robust foundation for subsequent MALDI imaging, enabling more accurate comparisons among regions and samples. Importantly, PFA fixation and agarose embedding preserved the native lipid distributions throughout the organ. The interpretation of the acquired MALDI data is further facilitated by automatic registration of the Allen mouse brain atlas, adding anatomical annotations and facilitating the construction of comprehensive whole-tissue molecular maps.

## Supplementary Material





## Notes

Identified lipids in the MALDI-MSI data set using lipidomics LC–MS/MS data (XLSX)
High spatial resolution comparison of Marinus MP-generated and fresh frozen brain sections using MALDI-MSI, multiplatform evaluation of the Marinus MP system for MALDI-MSI, and image alignment strategy (PDF)
