# Supplementary material for: Enabling High-Throughput Tissue Sectioning for Robust Lipid Mass Spectrometry Imaging
Source: Anal Chem. 2026 Sep 22;98(39):28629–37. doi: 10.1021/acs.analchem.6c03106 (PMC13647983; doi:10.1021/acs.analchem.6c03106)
Supplement: Supplementary file 1 [file ac6c03106_si_002.pdf]

Supporting Information: Enabling high-throughput tissue sectioning for robust lipid mass spectrometry imaging

Ruben Jacobs [1], Ritesh Chidambaram [2], Vincent Steinbacher [3], Chenwei Wei[2], Tim Ragan [2], Michiel Vandenbosch [1±] and Ron M.A. Heeren [1±*]

[1] The Maastricht MultiModal Molecular Imaging (M4I) institute, Division of Imaging Mass Spectrometry (IMS), Maastricht University, 6229 ER Maastricht, The Netherlands

[2] TissueVision 150 California St STE 303, Newton, MA 02458, The United States

[3] Institute of Molecular Immunology, School of Medicine and Health, Technical University of Munich (TUM), 81675 Munich, Germany

± contributed equally

** Corresponding author:*
Prof. Ron M.A. Heeren - *The Maastricht MultiModal Molecular Imaging (M4I) institute, Division of Imaging Mass Spectrometry (IMS), Maastricht University, 6229 ER Maastricht, The Netherlands*
r.heeren@maastrichtuniversity.nl
https://orcid.org/0000-0002-6533-7179

Authors:
Ruben Jacobs - *The Maastricht MultiModal Molecular Imaging (M4I) institute, Division of Imaging Mass Spectrometry (IMS), Maastricht University, 6229 ER Maastricht, The Netherlands*
ruben.jacobs@maastrichtuniversity.nl
https://orcid.org/0009-0002-5933-936X

Michiel Vandenbosch - *The Maastricht MultiModal Molecular Imaging (M4I) institute, Division of Imaging Mass Spectrometry (IMS), Maastricht University, 6229 ER Maastricht, The Netherlands*
m.vandenbosch@maastrichtuniversity.nl
https://orcid.org/0000-0002-0427-416X

Prof. Ron M.A. Heeren - *The Maastricht MultiModal Molecular Imaging (M4I) institute, Division of Imaging Mass Spectrometry (IMS), Maastricht University, 6229 ER Maastricht, The Netherlands*
r.heeren@maastrichtuniversity.nl
https://orcid.org/0000-0002-6533-7179

Timothy Ragan - *TissueVision, Inc. 150 California St STE 303, Newton, MA 02458, The United States*
tragan@tissuevision.com
https://orcid.org/0000-0001-6300-0858

Vincent Steinbacher - *Institute of Molecular Immunology, School of Medicine and Health, Technical University of Munich (TUM), 81675 Munich, Germany*
vincent.steinbacher@tum.de
https://orcid.org/0009-0007-2856-0471

Ritesh Chidambaram - *TissueVision, Inc. TissueVision 150 California St STE 303, Newton, MA 02458, The United States*
rchidambaram@tissuevision.com
https://orcid.org/0009-0001-1797-1191

Chenwei Wei - *TissueVision, Inc. TissueVision 150 California St STE 303, Newton, MA 02458, The United States*
cwei@tissuevision.com
https://orcid.org/0009-0000-1790-5082

Table of contents

Supplemental Figure S1: High spatial resolution comparison of Marinus MP-generated and fresh frozen brain sections using MALDI-MSI ------------------------------------- **S4**
Supplemental Figure S2. Multiplatform evaluation of the Marinus MP system for MALDI-MSI -- **S5**
Supplemental Figure S3: Image alignment strategy ---------------------------------- **S6**

Supplemental data S1: Identified lipids in the MALDI-MSI data set using lipidomics LC-MS/MS data

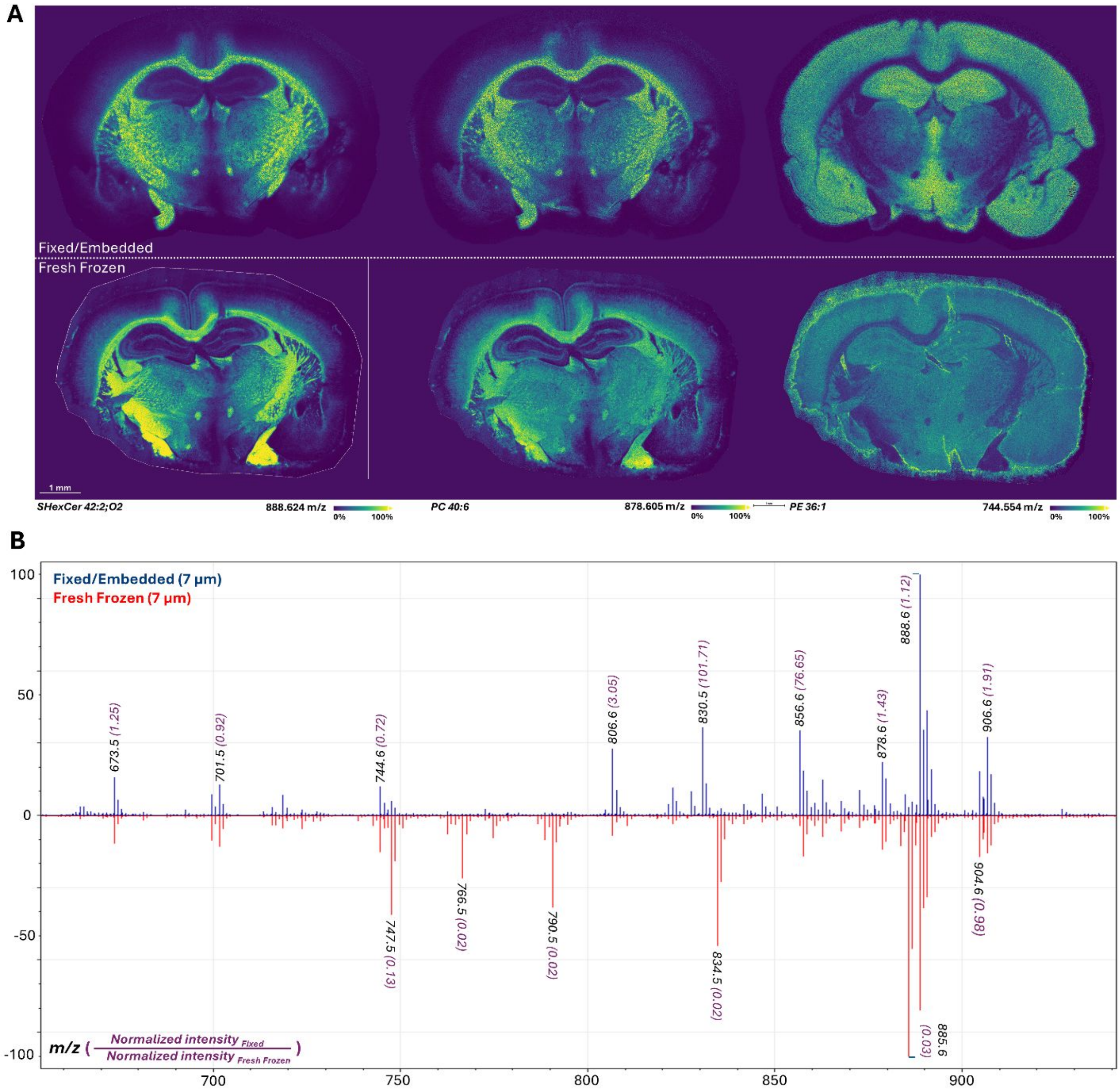

Supplemental Figure S1: High spatial resolution comparison of Marinus MP-generated and fresh frozen brain sections using MALDI-MSI

A. *High spatial resolution MALDI-MSI acquired on the timsTOF fleX at 7 µm pixel size of a PFA perfusion fixed and embedded mouse brain section generated by the Marinus MP system (top). High spatial resolution MALDI-MSI acquired on the timsTOF fleX at 7 µm pixel size of a fresh frozen non-fixed section (bottom). Both measurements were performed in negative ion mode.*

B. *Spectral comparison (lipid range) between fresh frozen and PFA fixed/embedded tissue. Most abundant species were annotated, and ratios are shown for comparison (ratio = normalized intensity fixed / normalized intensity fresh frozen).*

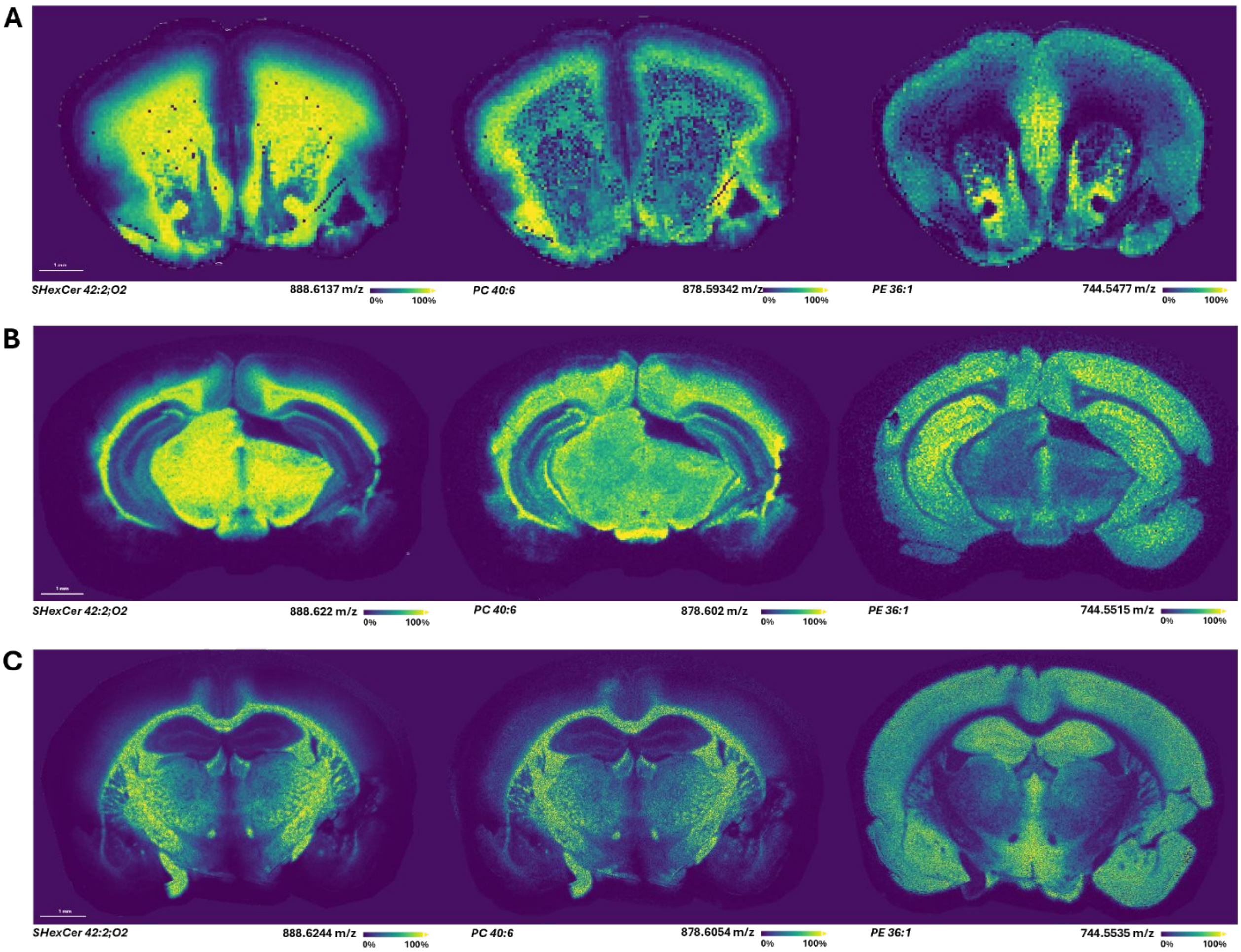

Supplemental Figure S2: Multi-platform evaluation of the Marinus MP-generated brain sections for MALDI-MSI at enhanced spectral and spatial resolution.

All measurements were performed in negative ion mode. Three distinct lipid species are shown across different MALDI applications.

A. *High mass resolution FT-ICR MALDI-MSI performed on a solariX instrument at 70 µm spatial resolution. Mass resolving power 354.000 at m/z 600.*

B. *TimsTOF fleX MALDI-MSI acquired from a non-conductive slide at 50 µm spatial resolution.*

C. *High spatial resolution MALDI-MSI acquired on the timsTOF fleX at 7 µm pixel size.*

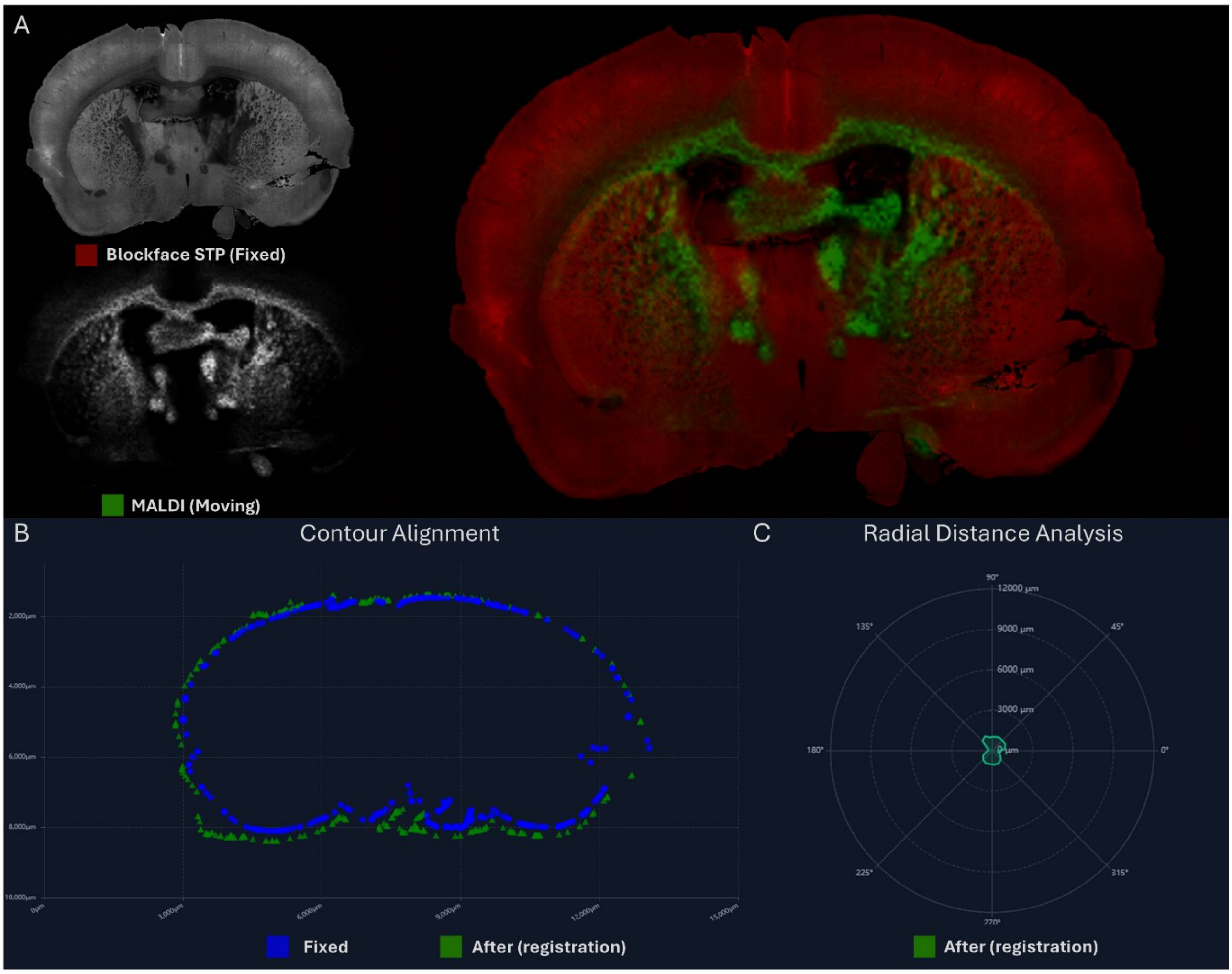

Supplemental Figure S3: Image alignment strategy.
A. *The moving dataset (MALDI) is registered to the fixed (STP) dataset using a series of preprocessing and Elastix non—rigid operations.*
B. *Fixed contour points are matched with moving contour points. The resulting mean residual distance from the matched pairs are shown.*
C. *Matched pairs (fixed-moving) are binned about the fixed image's center of mass to produce a per-sector distance profile, visualized using a radar chart.*
